# Augmentation of Pulmonary Epithelial Cell IL-8 Expression and Permeability by Pre-B-cell Colony Enhancing Factor

**DOI:** 10.1186/1476-9255-5-15

**Published:** 2008-09-22

**Authors:** Hailong Li, Peng Liu, Javier Cepeda, Deyu Fang, R Blaine Easley, Brett A Simon, Li Qin Zhang, Shui Qing Ye

**Affiliations:** 1 Department of Surgery, University of Missouri School of Medicine, Columbia, MO 65212, USA; 2 Department of Molecular Microbiology and Immunology, University of Missouri School of Medicine, Columbia, MO 65212, USA; 3 Department of Otolaryngology, University of Missouri School of Medicine, Columbia, MO 65212, USA; 4 Department of Anesthesiology and Critical Care Medicine, The Johns Hopkins University, Baltimore, MD 21205, USA; 5 Department of Medicine, The Johns Hopkins University, Baltimore, MD 21205, USA

## Abstract

**Background:**

Previous studies in our lab have identified Pre-B-cell colony enhancing factor (PBEF) as a novel biomarker in acute lung injury (ALI). The molecular mechanism of PBEF involvement in the pathogenesis of ALI is still incompletely understood. This study examined the role of PBEF in regulating pulmonary alveolar epithelial cell IL-8 expression and permeability.

**Methods:**

Human pulmonary alveolar epithelial cells (cell line and primary cells) were transfected with human PBEF cDNA or PBEF siRNA and then cultured in the presence or absence of TNFα. PBEF and IL-8 expression were analyzed by RT-PCR and Western blotting. In addition, changes in pulmonary alveolar epithelial and artery endothelial cell barrier regulation with altered PBEF expression was evaluated by an *in vitro *cell permeability assay.

**Results:**

Our results demonstrated that, in human pulmonary alveolar epithelial cells, the overexpression of PBEF significantly augmented basal and TNFα-stimulated IL-8 secretion by more than 5 to 10-fold and increased cell permeability by >30%; the knockdown of PBEF expression with siRNA significantly inhibited basal and TNFα-stimulated IL-8 secretion by 70% and IL-8 mRNA levels by 74%. Further, the knockdown of PBEF expression also significantly attenuated TNFα-induced cell permeability by 43%. Similar result was observed in human pulmonary artery endothelial cells.

**Conclusion:**

These results suggest that PBEF may play a vital role in basal and TNFα-mediated pulmonary inflammation and pulmonary epithelial barrier dysfunction via its regulation of other inflammatory cytokines such as IL-8, which could in part explain the role of PBEF in the susceptibility and pathogenesis of ALI. These results lend further support to the potential of PBEF to serve as a diagnostic and therapeutic target to ALI.

## Background

Acute lung injury (ALI) is characterized by pulmonary inflammation, non-cardiogenic edema, and severe systemic hypoxemia. Acute respiratory distress syndrome (ARDS) is the severe form of ALI [1,2]. One of the earliest manifestations of ALI is a diffuse intense inflammatory process and damage to both endothelial and epithelial cell barriers, resulting in marked extravasation of vascular fluid into the alveolar airspace [3]. A number of inflammatory cytokines including tumor necrosis factor-alpha (TNFα) and interleukin 8 (IL-8) can induce or aggravate the inflammation of endothelial and epithelial cells, leading to this barrier dysfunctions [4]. The mortality and morbidity of ALI/ARDS remain high since the etiology and molecular pathogenesis are still incompletely understood.

Our previous study, based on extensive microarray gene expression profiling in canine, murine, and human ALI, revealed pre-B-cell-colony-enhancing factor (PBEF) as a significantly upregulated gene [5]. Analysis of single nucleotide polymorphisms (SNPs) in the PBEF gene proximal promoter region indicated that a GC haplotype had a higher risk of ALI while a TT haplotype may have a lower risk of ALI [5]. Our findings were confirmed and extended by Bajwa et al [6], who showed that the PBEF T-1001G variant allele and related haplotype are associated with increased odds of developing ARDS and increased hazard of intensive care unit mortality among at-risk patients. In contrast, the C-1543T variant allele and related haplotype are associated with decreased odds of ARDS among patients with septic shock and better outcomes among patients with ARDS. In a mechanistic study, we found that PBEF is critically involved in thrombin-induced lung endothelial cell barrier dysregulation [7].

The objective of this study was to further elucidate the role of PBEF in pulmonary epithelial cell inflammation and barrier regulation since impaired alveolar epithelial fluid transport is also a characteristic feature in patients with ALI and has been associated with increased morbidity and mortality [4]. Using A549 human pulmonary alveolar epithelial cells and primary bronchial airway epithelial cells, we assessed the effect of PBEF knockdown with PBEF-specific silencing RNA (siRNA) and the effect of PBEF overexpression on TNFα-mediated IL-8 production and on cellular barrier function. Effect of the altered PBEF expression on basal or TNFα stimulated primary human pulmonary artery endothelial cells permeability was also examined to indirectly validate the similar results in the A549 cell line. Study of the role of PBEF in TNFα-mediated pulmonary cell IL-8 production and resultant barrier dysfunctions may help elucidate the molecular mechanisms underlying the role of PBEF in the susceptibility and pathogenesis of ALI.

## Methods

### Materials

Rabbit anti-human IL-8 polyclonal antibody (Cat. No. sc-7922, Santa Cruz, California, USA) and mouse anti-human β-actin monoclonal antibody (Cat. No. A1978) were obtained from Sigma-Aldrich (St. Louis, MO, USA). Rabbit anti-human PBEF polyclonal antibody was from Bethyl Laboratories, Inc. (Cat. No. A300-372A, Montgomery, TX, USA). Total mouse lung RNA was from Stratagene (Cat. No. 736511, La Jolla, CA, USA). Recombinant human TNFα (Cat. No. 210-TA) was from R&D Systems Inc. (Minneapolis, MN, USA). Superscript III Reverse Transcriptase (Cat. No. 18080044), Platinum Taq DNA polymerase (Cat. No. 10966018) was from Invitrogen (Carlsbad, CA, USA). Tricine was purchased from the Sigma-Aldrich (Cat. No. T0377, St. Louis, MO, USA). Sources of other key reagents are specified in the relevant text.

### Cell culture

Human A549 cells, a lung carcinomatous type II alveolar epithelial cell line, were obtained from ATCC (Cat. No. CCL-185™, Manassas, VA) and maintained in a Dulbecco's Modified Eagle's Medium supplemented with 10% fetal bovine serum, 2 mM glutamine, and penicillin/streptomycin. Primary human lung small airway epithelial cells (Cat. No. CC-2547) were obtained from Lonza (Walkersville, MD, USA) and maintained in a small airway epithelial cell basal medium (Cat. No. CC-3119) with Supplement & Growth factors (Cat. No. CC-4124). Primary human pulmonary artery endothelial cells (HPAEC, Cat. No. CC-2530) were obtained from Cambrex Bio Science Inc. (Walkersville, MD, USA) and maintained in EGM™-2 Endothelial Cell Medium-2 (Cat. No. CC-4176). All cells were cultured at 37°C in a humidified atmosphere of 5% CO_2_, 95% air. Cells from each primary flask were detached with 0.05% trypsin, resuspended in fresh culture medium, and seeded into 6-well plates for Western blot and RT-PCR analysis or seeded into the culture inserts for *in vitro *cell permeability assays.

### Transfection of PBEF siRNA into human A549 cells, primary human lung small airway epithelial cells and primary HPAEC

PBEF stealth siRNA was designed based on the human PBEF cDNA reference sequence (NM_005746.1) using the BLOCK-iT™ RNAi Designer (Invitrogen, Carlsbad, CA, USA). Using GFP-labeled non-specific siRNA, we first optimized the conditions for human A549 cells transfection and achieved >90% transfection efficiency using the Lipofectamine 2000 reagent (Invitrogen, Carlsbad, CA, USA). To transfect PBEF stealth siRNA into human A549 cells, cells were seeded for 24 h in the regular growth medium (without antibiotics) so that they would be 80–90% confluent at the time of transfection. For each transfection in 24-well plates, 50 pmol PBEF stealth siRNA was diluted in 50 μl Opti-MEM I without serum and gently mixed with 1 μl Lipofectamine 2000 diluted in the 50 μl Opti-MEM I (Invitrogen, Cat. 31985-062). After incubation for 15 min at room temperature, PBEF stealth siRNA and Lipofectamine 2000 complexes were added to each well. Cell culture plates were gently mixed by rocking back and forth. The amount of PBEF stealth siRNA and Lipofectamine 2000 were adjusted according to the different sizes of cell culture plates. Transfected cells were further incubated at 37°C for 24–48 h until treatment with TNFα before intended assays were carried out. Primary human lung small airway epithelial cells were similarly transfected. Transfection of PBEF siRNA into HPAEC was performed as previously described by Ye et al [7].

### Preparation and expression of the PBEF-overexpressing construct pCAGGS-hPBEF

A human PBEF (hPBEF) coding cDNA was amplified from A549 cell total RNA by RT-PCR using the following primer pair designed according to the reference human PBEF mRNA sequence (NM_005746.2): forward primer, 5'-TTAGAATTC**GCCACC**ATGCCTGCGGCAGAAGCC-3'and reverse primer, 5'-TTAGAATTCTTA***ATGGTGATGGTGATGATG***CAAATGATGTGCTGCTTCCAGTTC-3'. The regular bold letters indicate the optimized Kozac sequence. The bold italic letter part is His tag sequence. The underlined sequences are EcoRI adaptors. The amplified human PBEF cDNA was digested with EcoRI and subcloned into the unique EcoRI site of pCAGGS vector, which was provided by Dr. Deyu Fang (Department of Molecular Microbiology and Immunology, University of Missouri-Columbia). After the cloning, pCAGGS-hPBEF was sequence-verified. In this construct, human PBEF expression was driven by a chicken beta-actin/rabbit beta-globin hybrid promoter (AG) with an enhancer from the human cytomegalovirus immediate early promoter (CMV-IE). Overexpression of PBEF in A549 cells and HPAEC was carried out by a transient transfection of pCAGGS-hPBEF. Briefly, one day before transfection, A549 cells or HPAEC were plated in 6-well plate at 5 × 10^5 ^cells/well in 2 ml of growth medium without antibiotics. On the day of transfection, cells were at 95% confluence. For each well, 4 μg plasmid DNA was transfected using Lipofectamine 2000 according to the suppliers' instruction. Cell medium and cell lysate proteins were harvested at 48, 72 and 96 hours after the transfection for western blotting analyses of IL-8, PBEF and β-actin protein levels in A549 cells. pCAGGS-hPBEF or pCAGGS transfected HPAEC were used only for the assessment of cell permeability.

### Isolation of RNA and RT-PCR analysis

Total RNA was isolated from A549 cells with TRIZOL solution (Cat. No. 15596-018, Invitrogen, Carlsbad, CA, USA) according to the supplier's instructions. RT-PCR was performed using Invitrogen RNA PCR kit (Superscript III, 18080-044) with the following procedures: 1 μg total RNA was reverse transcribed with random primer at 50°C for 1 h followed by 70°C for 15 min and 4°C for 5 min in a 20 μl reaction volume. Each PCR reaction from the cDNA template (2 μl RT product) was performed using gene specific primers (Table 1) at 94°C for 3 min, then 32 cycles at 94°C for 1 min, 55°C for 1 min and 72°C for 1 min, followed by 72°C for 7 min for the final extension. β-actin was used as a house-keeping gene control. PCR products were separated on a 1.5% agarose gel and stained by Ethidium Bromide (0.5 μg/ml). The band image was acquired using an Alpha Imager and analyzed by the AlphaEase™ Stand Alone Software (Alpha Innotech Corp., San leandro, CA, USA).

**Table 1:** Primers and products sizes

Products	5' Primers	3' Primers	Size (bp)	Accession No.
IL-8	ATGACTTCCAAGCTGGCCGT	CCTCTTCAAAAACTTCTCCACACC	297	NM_000584
PBEF	AAGCTTTTTAGGGCCCTTTG	AGGCCATGTTTTATTTGCTGACAAA	319	NM_005746
β-actin	CAAACATGATCTGGGTCATCTTCTC	GCTCGTCGTCGACAACGGCTC	487	NM_001101

### Western blotting

Western blot analysis was performed following the protocol of Bio-Rad Company. Briefly, after washing with PBS, cells were lysed with 500 μl of cell lysis buffer containing 10 mM Tris (pH 7.4), 1% Triton X-100, 0.5% Nonidet P-40, 150 mM NaCl, 1 mM EDTA, 0.2 mM EGTA, 0.2 mM vanadate, 0.2 mM PMSF, and 0.5% protease inhibitor cocktail. Total cell lysates were cleared by centrifugation and boiled with the same amount of 4× SDS sample buffer for 5 min. Total protein of cell lysates was quantified using the BCA Protein Assay Kit (Pierce Biotechnology, Inc., Rockford, IL, USA). An equal amount of total protein from each sample was then subjected to 16.5% Tris/tricine polyacrylamide gel electrophoresis. The separated proteins were transferred to PVDF membranes by electrotransfer. The blots were subsequently blocked with 5% bovine serum albumin in PBS containing 0.1% Tween 20 (TBS-T) at room temperature for 1 h and then incubated at 4°C overnight with primary antibodies of interest. After washing three times for 10 min with TBS-T, the membrane was incubated with horseradish-peroxidase-linked secondary antibodies of interest at room temperature for 1 h. The blots were then visualized with the ECL Western blot detection system (Cat. No. RPN2106, Amersham Bosciences, Buckinghamshire, UK). The same membrane was re-probed with an anti-human β-actin antibody. β-actin was used as an internal control. Band density on Western blot images was used as a measure of assayed protein level. The band image was acquired using an Alpha Imager and analyzed by the AlphaEase™ Stand Alone Software.

### In Vitro Cell Permeability Assay

*In Vitro *Cell Permeability Assay was carried out according to the protocol of the *CHEMICON in vitro *Vascular Permeability Assay kit (Cat. No. ECM640, Millipore, Billerica, MA, USA). Briefly, cells were seeded to the culture inserts of permeability chambers (1.0 × 10^6 ^cells/ml) that were coated with collagen. Then, cells were incubated in 37°C and 5% until a monolayer was formed. After TNFα (Cat. No. 636-R1, R&D systems, Minneapolis, MN, USA) was added, cells were incubated for another 18 hours at 37°C and 5% CO_2 _in the tissue culture incubator. Finally, 150 μl of FITC-Dextran was added to each insert for 5 min at room temperature, and then 100 μl of the solution in the bottom chamber was transferred to a 96-well plate. The plate was read in a TriStar Multimode Reader (LB 941, Berthold Technologies GnbH & Co. KG, Bad Wildbad, Germany) at wavelengths of 485 and 530 nm. Reagent control wells were treated with basal medium and growth medium only. Blank inserts without cells plated were also included as controls.

### Statistical analysis

Statistical analyses were performed using SigmaStat (ver 3.5, Systat Software, Inc., San Jose, CA, USA). Results are expressed as mean ± standard deviation (SD) of four samples from at least two independent experiments. Stimulated samples were compared with controls by unpaired Student's t test. P < 0.05 was considered statistically significant.

## Results

### Dose-response and time-course of TNFα induced IL-8 protein expression in A549 cells

**Figure 1:**
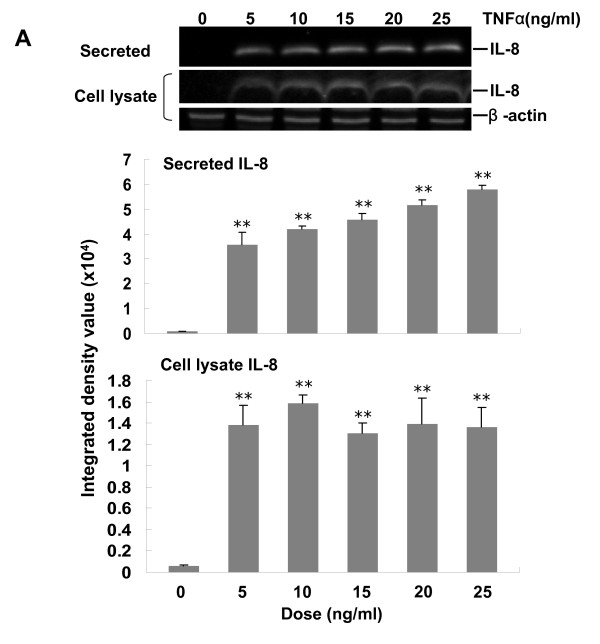
**Dose-response and time-course of TNFα-induced IL-8 protein expression in A549 cells**. *A*. Dose-response. The top panel illustrates a typical western blotting image of IL-8 and β-actin protein detections. After starving for serum overnight, A549 cells were stimulated with different doses of TNFα as indicated for 24 hours. Equal amount of total protein from each sample was separated by 16.5% SDS-PAGE and immunodetected by the western blotting using anti-human IL-8 or β-actin antibodies. Middle panel-Quantitation of secreted IL-8 level by densitometric analysis. Results from each group are presented as mean ± SD of 4 samples from two separate experiments. Bottom panel-Quantitation of cell lysate IL-8 level by densitometric analysis. **p < 0.01 vs. control (0 ng.ml TNFα). *B*. Time-course. Top panel-Representative western blotting image of IL-8 and β-actin protein detections. After starving for serum overnight, A549 cells were stimulated with TNFα (15 ng/ml) for different time (h) as indicated. Middle panel-Quantitation of secreted IL-8 protein level by densitometric analysis. Bottom panel-Quantitation of cell lysate IL-8 protein level by densitometric analysis. *p < 0.05 and **p < 0.01 vs 0 dosage of TNFα or 0 h control.

In order to examine the role of PBEF in human pulmonary alveolar epithelial cell inflammation, we began by quantifying TNFα induced IL-8 protein expression within A549 cells. We first determined the dose-response and time-course of TNFα induced IL-8 protein expression in A549 cells in our experimental conditions. The results (Figure 1, panel A) demonstrate that TNFα treatment for 24 h significantly induced IL-8 secretion in A549 cells in a dose-dependent manner up to the highest tested concentration of TNFα (25 ng/ml). Within the cell lysate, IL-8 level was also increased with TNFα treatment but was not dose-dependent within the tested dose range. Secreted IL-8 levels was also significantly increased in a time-dependent manner after TNFα (15 ng/ml) treatment compared with control group (Figure 1, panel B), especially at 6, 18, and 24 h time points. Again, cell lysate IL-8 levels were increased with treatment but not in a time dependent manner. These data indicate that TNFα significantly increased IL-8 secretion in dose-dependent and time-dependent manners in A549 cells in our experimental conditions. The 24 h time point and 15 ng/ml of TNF α dosage were selected for subsequent experiments.

### Dose-response and time-course of TNFα induced PBEF protein expression in A549 cells

**Figure 2:**
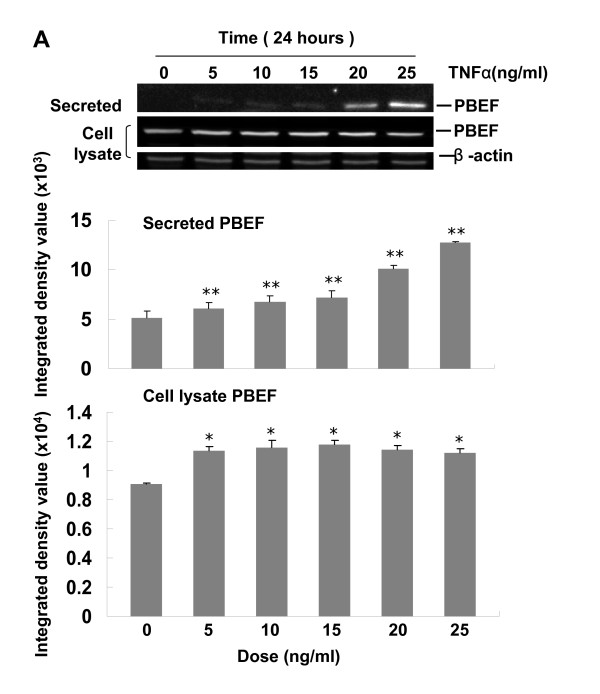
**Dose-response and time-course of TNFα induced PBEF protein expression in A549 cells**. *A*. Dose-response. The top panel is a typical western blotting image of PBEF and β-actin protein detections. After starving for serum overnight, A549 cells were stimulated with different doses of TNFα, as indicated, for 24 hours. Equal amount of total cell lysate protein from each sample was separated by 10% SDS-PAGE and immunodetected by the western blotting using anti-human PBEF or β-actin antibodies. Middle panel-Quantitation of secreted PBEF level by densitometric analysis. Results from each group are presented as mean ± SD of 4 samples from two separate experiments. The bottom panel is the quantitation of cell lysate PBEF protein level by densitometric analysis. *B*. Time-course. Top panel-Representative western blotting image of PBEF and β-actin protein detections. After starving for serum overnight, A549 cells were stimulated with TNFα (15 ng/ml) for different time (h) as indicated. Lower panel-Quantitation of cell lysate PBEF protein level by densitometric analysis. *p < 0.05 and **p < 0.01 vs 0 dosage of TNFα or 0 h control.

After a pilot experiment provided evidence that PBEF expression could be induced by TNFα treatment in A549 cells (data not shown), we sought to determine the dose-response and time-course to optimize the experimental conditions of TNFα induced PBEF expression in A549 cells. In this dose-response experiment (Figure 2, panel A), our results show that secreted PBEF level was increased in a dose-dependent manner and cell lysate PBEF expression was significantly increased compared to control at all dose treatments of TNFα tested [15 ng/ml TNFα treatment vs. control: 1.17 ± 0.02 vs. 0.9 ± 0.005, n = 4, p < 0.05]. In the time-course experiment (Figure 2, panel B), secreted and intracellular PBEF protein expression continued to increase over the 24 h of TNFα treatment [TNFα treatment vs. control at 24 h: 1.56 ± 0.04 vs. 0.837 ± 0.03, n = 4, p < 0.01]. The dose-response and time-course of TNFα mediated PBEF expression in A549 cells are similar to those of IL-8.

### TNFα induction of IL-8 and PBEF expression at their mRNA levels in A549 cells

**Figure 3:**
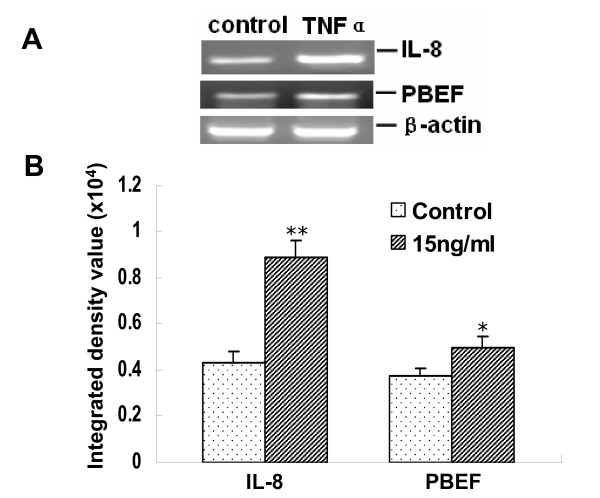
**Effect of TNFα treatment on the mRNA expression of IL-8 and PBEF in A549 cells**. *A*. A representative gel image of IL-8, PBEF and β-actin mRNA detections. After starving for serum overnight, A549 cells were stimulated with TNFα (15 ng/ml) for 4 h. Total cell RNA was reverse-transcribed, amplified by PCR using the gene specific primers (Table 1), separated by 1.5% agarose electrophoresis, and visualized with ethidium bromide. *B*. Quantitation of IL-8 and PBEF mRNA levels by densitometric analysis. Results from each group are presented as mean ± SD of 4 samples from two separate experiments. *p < 0.05 and **p < 0.01 vs 0 dosage of TNFα or control.

To investigate whether TNFα augments IL-8 and PBEF expression at their mRNA levels in A549 cells, we performed a semi-quantitative RT-PCR analysis of IL-8 and PBEF mRNA levels in TNFα induced A549 cells. As presented in Figure 3, IL-8 and PBEF mRNA levels in TNFα treated A549 cells were significantly increased compared to the control groups (IL-8 mRNA expression: 0.89 ± 0.04 vs. 0.42 ± 0.05, n = 4, p < 0.01; PBEF mRNA expression: 0.5 ± 0.04 vs. 0.37 ± 0.025, n = 4, p < 0.05; respectively).

### Knockdown of PBEF protein and mRNA expression by PBEF stealth siRNA in A549 cells

**Figure 4:**
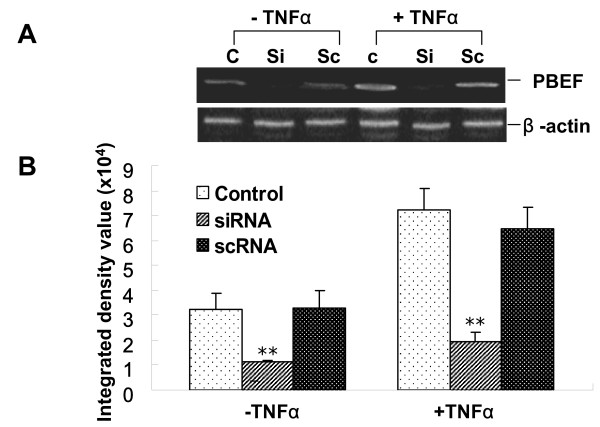
**PBEF stealth siRNA-mediated silencing of PBEF protein and mRNA expression in A549 cells**. *A*. Representative western blotting image of PBEF protein detections. After starving for serum overnight, A549 cells were transfected with the control (C), scrambled siRNA (Sc), or 50 nmolar PBEF stealth siRNA (Si) for 48 h before treatment without or with TNFα (15 ng/ml) for 24 hours. Cell lysate PBEF and β-actin protein were immunodetected as described. *B*. Quantitation of PBEF protein levels by densitometric analysis. A549 Cell lysate PBEF protein levels in various treatments were quantified by densitometric analysis. **p < 0.01. *C*. A representative RT-PCR gel image of PBEF and β-actin mRNA detections. A549 cells were grown and transfected with the PBEF siRNA and other controls as described above and then treated without or with TNFα (15 ng/ml) for 4 hours. Total cell RNA was reverse-transcribed, amplified by PCR using gene specific primers (Table 1), separated by 1.5% agarose electrophoresis and visualized with ethidium bromide. *D*. Quantification of PBEF mRNA level by densitometric analysis. **p < 0.01 vs. control.

We first performed a pilot experiment to demonstrate that PBEF siRNA could in fact knock down PBEF expression in A549 cells before determining the optimal time course and dose response of PBEF stealth siRNA for the inhibition of PBEF protein expression in A549 cells (data not shown). The 48 h time point and 50 nm dosage were selected for the intended experimentation. In the baseline condition without the TNFα treatment (Figure 4, Panels A and B), PBEF siRNA significantly knocked down PBEF protein expression in A549 cells [siRNA vs. control: 1135 ± 33 vs. 3253 ± 624, n = 4, p < 0.01]. Scrambled RNA (scRNA) and reagent alone had no effect on PBEF protein expression. PBEF-specific siRNA had no effect on the protein expression level of β-actin, a house-keeping gene serving as a control. In the + TNFα treatment group (Figure 4, Panels A and B), PBEF protein level in the scRNA group had no significant change (scRNA vs. control: 7246 ± 856 vs. 6440 ± 889, n = 4, NS), while PBEF protein level in the siRNA group was significantly lower than in the control (siRNA vs. control: 1926 ± 415 vs. 7246 ± 856, n = 4, p < 0.01). Figure 4, panels C and D, illustrates the expected effect of PBEF siRNA on significantly inhibiting PBEF mRNA expression in both the baseline and treated groups. These results indicate that PBEF-specific siRNA can significantly reduce both PBEF protein and mRNA production in both baseline and TNFα activated A549 cells.

### PBEF silencing attenuated TNFα-induced increases in IL-8 secretion and IL-8 mRNA expression within A549 cells

**Figure 5:**
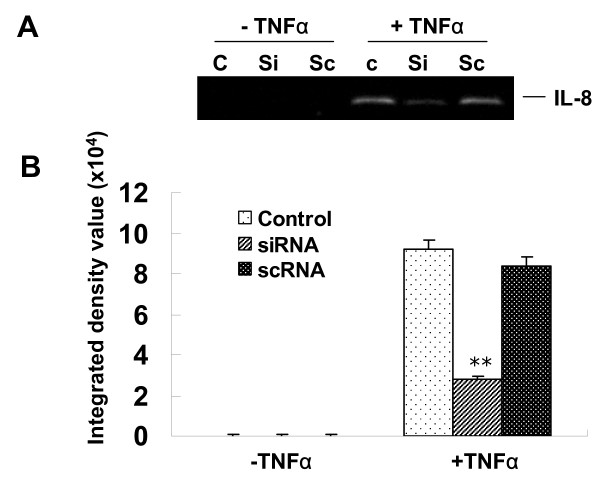
**Effects of PBEF knock down on IL-8 protein & mRNA expression in A549 cells (A-D) and in lung primary small airway epithelial cells (E-F)**. *A*. Representative western blotting image of secreted IL-8 protein detections. After starving for serum overnight, A549 cells were transfected with the vehicle control (C), scrambled siRNA (Sc), or PBEF stealth siRNA (Si) for 48 h before treatment without or with TNFα (15 ng/ml) for 24 hours. *B*. Quantitation of secreted IL-8 protein levels by densitometric analysis. **p < 0.01 vs. control. *C*. Representative RT-PCR gel image of IL-8 and β-actin mRNA detections. A549 cells were grown and transfected with the PBEF siRNA and other controls as described above before treatment without or with TNFα (15 ng/ml) for 4 hours. Total cell RNA was reverse-transcribed, amplified by PCR using gene specific primers (Table 1), separated by 1.5% agarose electrophoresis and visualized with ethidium bromide. *D*. Quantitation of IL-8 mRNA level by densitometric analysis. **p < 0.01 vs. control. *E*. Representative western blotting images of cell lysate PBEF and secreted IL-8 protein detections. Lung primary small airway epithelial cells were transfected with control (C), PBEF stealth siRNA (Si), or scrambled RNA (Sc) for 48 h before treatment without or with TNFα (10 ng/ml) for 12 hours. PBEF in cell lysate and secreted IL-8 protein were immunodetected as described in Figures 1 and 2. *F*. Quantitation of IL-8 and PBEF protein levels by densitometric analysis. **p < 0.01 vs. control.

Based on an efficient knock down of PBEF in A549 cells with PBEF siRNA, we next evaluated the effect of PBEF knockdown on the TNFα-induced increases in IL-8 secretion and IL-8 mRNA level from A549 cells. In Figure 5 (panels A and B) PBEF silencing significantly decreased IL-8 secretion from A549 cells in TNFα-stimulated conditions compared to the control group (siRNA vs. control: 2807.69 ± 161.2 vs. 9178.58 ± 512.64, n = 4, p < 0.01), while secreted IL-8 level in the scRNA group had a small and statistically insignificant change relative to the control group (scRNA vs. control: 8808.37 ± 400.52 vs. 9178.58 ± 512.64, n = 4, NS). Further, Figure 5 (panels C and D) demonstrates that PBEF silencing decreased IL-8 mRNA expression levels in the baseline (-TNFa) group (siRNA vs. control: 886.17 ± 190 vs. 3718.27 ± 360, n = 4, p < 0.01) as well as the treatment (+TNFα) group (siRNA vs. control: 2128.78 ± 96 vs. 3255.42 ± 107, n = 4, p < 0.05). These results indicate that PBEF is involved in IL-8 expression and secretion under both baseline and TNFα-stimulated conditions within the A549 cells.

### PBEF silencing attenuated TNFα-induced increases in IL-8 secretion and PBEF protein expression in lung primary small airway epithelial cells

To further confirm the above results, we also investigated PBEF effects on TNFα-induced IL-8 secretion in primary human lung small airway epithelial cells. In Figure 5 (panels E and F), PBEF silencing is shown to decrease IL-8 secretion in TNFα-stimulated conditions (siRNA vs. control: 10609.50 ± 4086.65 vs. 28801.42 ± 1235.48, n = 4, p < 0.01). In contrast, scRNA demonstrated no difference from control. These results indicate that PBEF is also involved in IL-8 expression and secretion in TNFα induced conditions in primary human lung small airway epithelial cells.

### PBEF over-expression augmented IL-8 secretion from A549 cells

**Figure 6:**
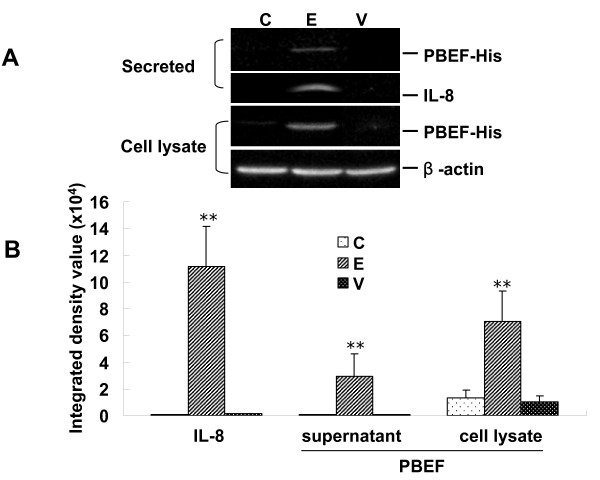
**Effects of PBEF overexpression on IL-8 secretion and PBEF production in A549 cells**. *A*. Representative western blotting image of PBEF and IL-8 protein detections. After starving for serum overnight, A549 cells were transfected with control (C), overexpressing construct pCAGGS-hPBEF (E) or vector pCAGGS (V) for 48 hours. Medium IL-8 and PBEF, Cell lysate PBEF and β-actin proteins were immunodetected as described in Figure 1 and Figure 2. *B*. Quantitation of PBEF and IL-8 protein levels by densitometric analysis. **p < 0.01 vs. control. C. representative western blotting image of PBEF and IL-8 protein detections with or without TNFα (15 ng/ml) treatment. A549 cells were transfected with control (C), pCAGGS-hPBEF (E) or vector pCAGGS (V) for 48 hours, and then stimulated by TNFα (15 ng/ml) for another 12 hours. Medium IL-8 and PBEF, cell lysate PBEF and β-actin proteins were immunodetected as described in above. D. quantitation of PBEF and IL-8 protein levels by densitometric analysis. **p < 0.01 vs. control.

Since PBEF silencing could decrease TNFα-induced IL-8 secretion from A549 cells, the next experiments evaluated whether PBEF over-expression would augment IL-8 secretion from A549 cells. Similarly treated non-transfected A549 cells served as the control. In Figure 6 (panels A and B), the over-expression of PBEF in A549 cells transiently transfected with pCAGGS-hPBEF was achieved in both the secreted PBEF levels (transfected vs. control: 29712.81 ± 16259.85 vs. 948.75 ± 136.7, n = 4, p < 0.01) and in cell lysate PBEF levels (transfected vs. control: 70666.3 ± 22445.3 vs. 13519.15 ± 5745.44, n = 4, p < 0.01). PBEF over-expression significantly increased IL-8 secretion from A549 cells compared to control group (transfected vs. control: 111548.4 ± 84104.5 vs. 1034 ± 212.5, n = 4, p < 0.01). These findings suggest that PBEF has a direct effect on IL-8 secretion from A549 cells. Further, Figure 6 (panels C and D) demonstrates that PBEF over-expression also significantly augmented TNFα-induced IL-8 secretion from A549 cells compared to control group (transfected vs. control: 604423.1 ± 82477.6 vs. 350227.4 ± 19794.1) and IL-8 production in cell lysate in A549 cells compared to control group (transfected vs. control: 420519.4 ± 49539.8 vs. 224406.9 ± 45849.1). Those result further confirmed that PBEF has a very important role in TNFα-induced IL-8 secretion in A549 cells.

### PBEF expression affected cell permeability in A549 cells and human pulmonary artery endothelial cells (HPAEC)

**Figure 7:**
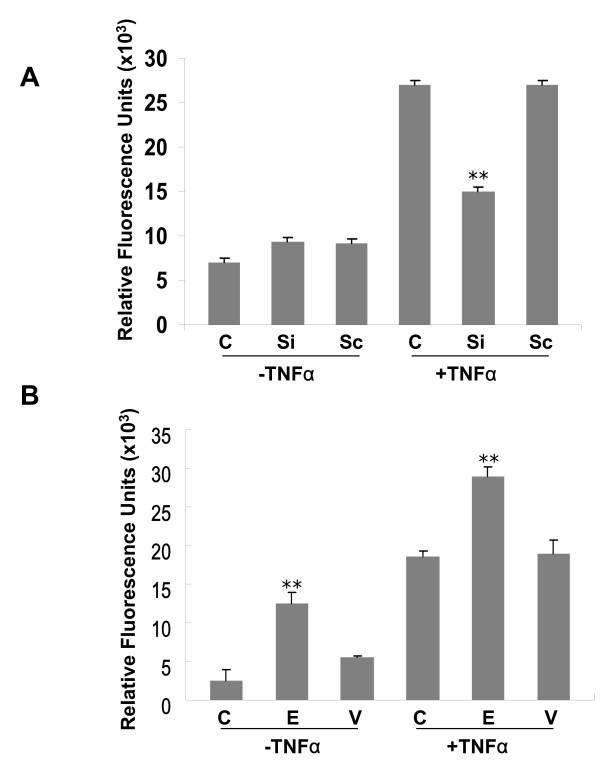
**Effects of PBEF expression on in vitro cell permeability of A549 cells and HPAEC**. A549 cells or HPAEC cells were seeded onto the culture inserts of permeability assay chambers and grown for 24 h before being transfected with the vehicle control (C), PBEF stealth siRNA (Si) or scrambled RNA (Sc), pCAGGS-hPBEF (E) or pCAGGS (V) for 48 h before treatment without or with 15 ng/ml TNFα. Then, the FITC-Dextran reagent was added to each insert for 5 min at room temperature. Leakage of FITC-Dextran into the bottom chamber was assayed as described in the Methods. *A&C*. Effects of PBEF underexpression on *in vitro *cell permeability of A549 cells and HPAEC; *B&D*. Effects of PBEF overexpression on *in vitro *cell permeability of A549 cells and HPAEC. **p < 0.01 vs. control.

To assess whether PBEF expression affected lung alveolar epithelial cell and HPAEC permeability, we performed *in vitro *monolayer cell permeability assays in A549 cells and HPAEC transfected with PBEF stealth siRNA or pCAGGS-hPBEF in the presence and absence of TNFα treatment. Controls were either scRNA or reagent vehicle without the vector, respectively. In Figure 7A, PBEF siRNA significantly decreased TNFα-induced permeability in A549 cells compared to the TNFα treated controls (siRNA vs. control: 15476.09 ± 577.35 vs. 27065 ± 563.04, n = 4, p < 0.01). Scrambled RNA had no effect on the TNFα-induced cell permeability in A549 cells. Under baseline (-TNFα) conditions, no differences in cell permeability were detected. These results indicate PBEF siRNA significantly attenuated the TNFα-induced barrier-disruption within the epithelial cells. To validate our observation in A549 cells, we performed the same experiment in a primary HPAEC. As displayed in Figure 7C, similar observations were obtained in HPAEC, whose cell permeability level in the PBEF siRNA group was significantly decreased compared to the TNFα alone treatment (13325 ± 1527.2 vs. 30850 ± 937.5 relative fluorescence units, n = 4, **P < 0.01). Scrambled RNA had no effect on the TNFα induced cell permeability in both A549 cells and HPAEC (Figure 7C). In Figure 7B, PBEF overexpression significantly promoted TNFα-induced A549 cell permeability level in the pCAGGS-hPBEF transfected group compared to the control groups (overexpression vs. control: 29156.09 ± 113.57 vs. 18570.4 ± 84.85, n = 4, p < 0.01). Similar observations were obtained in HPAEC cells (Figure 7D), whose cell permeability level in the PBEF overexpression group is significantly increased compared to the TNFα alone treatment (36955 ± 306.4 vs. 26331 ± 300.69 relative fluorescence units, n = 4, **P < 0.01). Scrambled RNA had no effect on the TNFα induced cell permeability in both A549 and HPAEC cells (Figure 7A and 7C). Even under baseline (-TNFa) conditions, overexpressing PBEF significantly increased cell permeability in A549 cells (over-expression vs. control: 12506.14 ± 141.42 vs.2537.4 ± 154.27, n = 4, p < 0.01) and HPAEC (22336.45 ± 70.71 vs.13675.4 ± 117.85 relative fluorescence units, n = 4, *P < 0.05). These results indicate that overexpression of PBEF augmented *in vitro *lung epithelial cell permeability under both the baseline and TNFα-induced conditions.

## Discussion

Our findings demonstrate that the modulation of PBEF expression resulted in parallel changes in TNFα-mediated IL-8 production and secretion as well as alveolar epithelial cell permeability. These results provide new insight into the role of PBEF in the inflammatory pathways and functional abnormalities associated with ALI.

There is considerable experimental and clinical evidence that pro-inflammatory cytokines play a major role in the pathogenesis of ALI/ARDS from inflammatory causes, such as sepsis, pneumonia, aspiration, and shock [8], as well as from mechanical stress [9-11]. The landmark ARDSnet clinical trial found that a lung-protective ventilatory strategy reduces mortality by 22% in patients with ALI, a result that in part may be ascribed to the marked reduction in the concentration of pro-inflammatory cytokines released into the airspaces of the injured lung [12,8]. TNFα and IL-8 are among important early mediators of ALI [4]. It has been shown that TNFα is present in increased amounts in the bronchoalveolar lavage fluid (BALF) of patients with ARDS [13], in the serum during the onset of sepsis-induced lung injury [14], and acutely increases in both serum and BALF when changing from a lung protective to non-protective ventilation strategy [9]. Increasing TNFα biological activity has been demonstrated over the first week of ARDS and there are direct relationships between the molar ratio of TNFα/soluble TNF-receptor in the BALF and severity of ALI (lung compliance and severity of hypoxemia) [13]. The role of TNFα in pulmonary pathophysiology has been well studied, and includes induction of cellular inflammatory reactions, enhancement of oxidative stress, and increased expression of various proinflammatory molecules [15].

Among the TNFα induced pro-inflammatory cytokines, IL-8 is regarded as one of the most important mediators in the pathogenesis of ARDS. In BALF, IL-8 levels were significantly increased in patients with ARDS and correlated with the development of ARDS in at-risk patients [16,17]. IL-8 has been identified as one of biomarkers of ALI/ARDS mortality [18]. In fact, IL-8 was first purified and molecularly cloned as a neutrophil chemotactic factor from lipopolysaccharide-stimulated human mononuclear cell supernatants [19]. Since then, studies of models of acute inflammation have established IL-8 as a key mediator in neutrophil mediated acute inflammation [20]. In acid aspiration- and endotoxemia-induced ARDS in rabbits, IL-8 is produced in the lungs [21,22]. In both models, the abrogation of IL-8 activity reduces neutrophil infiltration as well as tissue damage. It was demonstrated that TNFα mediated IL-8 production can suppress neutrophil apoptosis [23] and thus potentially prolong neutrophil migration into the lungs and damage to lung tissues, including alveolar epithelial barrier function [24].

Our data in this study indicate that TNFα significantly induces PBEF expression at both the mRNA and protein levels in A549 cells (Figures 2, 3, 4, 5), suggesting that PBEF may be an intermediate target of TNFα involved in the inflammatory process during the pathogenesis of ALI. The knockdown of PBEF expression by PBEF siRNA significantly blunted TNFα-stimulated IL-8 secretion and its production in A549 cells (Figures 6, 7), and PBEF-overexpression augmented IL-8 secretion from A549 cell (Figure 7). These results support the concept that PBEF may be an inflammatory signal transducer of TNFα or other inflammatory stimuli to regulate the synthesis of IL-8 or other inflammatory cytokines. These conclusions can be corroborated by evidence in non-lung tissue studies. PBEF silencing has been shown to prevent the suppression of neutrophil apoptosis caused by TNFα, IL-8 and other mediators [25]. In addition, PBEF gene expression is up-regulated in severely infected fetal membranes [26]. Inflammatory stimuli in fetal membranes inducing NF-κB and AP-1 have been shown to up-regulate PBEF [27]. The recombinant human PBEF treatment of amnion-like epithelial cells and fetal membrane explants significantly increased IL-6 and IL-8 expression [28]. Considered together, PBEF could play a critical role as an inflammatory cytokine during the pathogenesis of ALI.

Both endothelial and epithelial injuries were observed in earlier ultrastructural studies of the lung in patients dying with ALI secondary to sepsis [29,30]. Increased vascular permeability and endothelial injury contribute to the profound pathophysiological derangements in ALI [31,32]. If the alveolar epithelium is also damaged, the change in both endothelial and epithelial permeability could lead to major alveolar flooding with high-molecular-weight proteins, with prolonged changes in gas exchange, altered compliance and a much higher likelihood of disordered repair [33]. Normally, the lung alveolar epithelium forms an extremely tight barrier that restricts the movement of proteins and liquid from the interstitium into the alveolar spaces. In ALI, impaired alveolar epithelial function in the lungs is a marker of poor outcome in ALI [34]. A number of inflammatory cytokines, including TNFα and IL-8, can induce or aggravate the inflammation of endothelial and epithelial cells, leading to their barrier dysfunctions [35]. In this study we have demonstrated (Figure 7) that the TNFα stimulated increased in A549 cell permeability is significantly attenuated by the knockdown of PBEF expression by PBEF siRNA. Overexpression of PBEF increased A549 cell permeability in both basal and TNFα induced conditions. These results suggest that PBEF may have an important role in epithelial cell barrier regulation. A549 cells are an alveolar Type II tumor epithelial cell line. While the type II epithelial cell only covers 7% of the alveolar surface area, it constitutes 67% of the epithelial cell number within the alveoli [36], pointing to its biochemical importance. The permeability increase of A549 cells in the TNFα-stimulated condition involves both paracellular permeability, with gap formation visualized by actin cytoskeleton staining, and basement membrane permeability [37]. Since A549 cells usually do not form a regular monolayer, we went to examine whether a similar effect can be observed in a primary HPAEC, which forms a typical monolayer. Indeed, we found that PBEF expression similarly affected the HPAEC permeability (Figure 7).

While an overexpression of PBEF results in an increased basal permeability, knocking down PBEF expression failed to alter the basal permeability both in A549 cells and HPAEC. It is possible that in our study, transfection of pCAGGS-PBEF engendered at least 3 fold higher PBEF protein expression, but transfection of PBEF siRNA only resulted in about 60% decrease of PBEF level, which might explain the subdued effect of the latter in the basal level. TNFα mediated effect may be partly due to its induction of IL-8 production. IL-8 has been demonstrated to induce actin fiber rearrangement and intercellular gap formation in endothelial cells [38]. IL-8 could have a similar effect on epithelial barrier function. Our previous study found that reductions in PBEF protein expression significantly attenuated endothelial barrier dysfunction induced by the potent edemagenic agent, thrombin, reflected by reductions in transendothelial electric resistance [7]. Furthermore, reductions in PBEF protein expression blunted thrombin-mediated increases in Ca^2+ ^entry, polymerized actin formation, and myosin light chain phosphorylation [7], events critical to the thrombin-mediated permeability response [32]. Whether similar mechanisms account for the attenuating effects of PBEF knockdown on TNFα-induced epithelial and endothelial cell barrier dysfunction remains to be elucidated in future studies.

## Conclusion

In summary, this study demonstrated that TNFα significantly induced PBEF and IL-8 expression at both the mRNA and protein levels in A549 cells. PBEF-overexpression augmented IL-8 secretion and epithelial cell permeability in A549 cells, and the knockdown of PBEF expression by PBEF siRNA significantly blunted TNFα-stimulated IL-8 secretion and mRNA production and attenuated TNFα-induced cell permeability of A549 cells. Since inflammation and increased permeability is a hallmark of the pathogenesis of ALI, these results suggest that PBEF may play a critical role as an inflammatory cytokine in the dysregulation of alveolar epithelial cell barriers in the pathogenesis of ALI. These results lend further support to the potential of PBEF to serve as a diagnostic and therapeutic target in future studies of lung injury.

## Competing interests

The authors declare that they have no competing interests.

## Authors' contributions

HL, PL, JC, DF, RE carried out all studies and statistical analyses, and drafted the manuscript. BS, LZ and SY conceived of the study, and participated in its design and coordination. All authors read and approved the final manuscript.
